# Anæsthesia of Drunkenness Recommended

**Published:** 1876-08

**Authors:** 


Anæsthesia of Drunkenness Recommended.—Prof. Lynk, of Terre Haute, Indiana, announces in the Cincinnati *Lancet and Observer* the wonderful discovery that the best of all anæsthetics is alcohol, drank to the extent of producing coma. Several cases are described in illustration. One was that of a lady who had a "tumor on her side." She was prepared for its removal by taking two ounces of brandy every half hour till four doses had been imbibed. Half an hour after the last dose he found her sleeping and quite unconscious, and proceeded to remove the tumor. The operation lasted fifteen minutes, during which she moaned and talked unintelligibly. She vomited once, as she had done once or twice before his arrival. The next day she was sitting up, feeling quite well. He is so delighted with his discovery that he predicts the universal adoption of it within ten years.—*Pacific Med and Surg. Jour.*

